# Remote Sensing and Ecological Variables Related to Influenza A Prevalence and Subtype Diversity in Wild Birds in the Lluta Wetland of Northern Chile

**DOI:** 10.3390/v15061241

**Published:** 2023-05-25

**Authors:** Soledad Ruiz, Pablo Galdames, Cecilia Baumberger, Maria Antonieta Gonzalez, Camila Rojas, Cristobal Oyarzun, Katherinne Orozco, Cristian Mattar, Pamela Freiden, Bridgette Sharp, Stacey Schultz-Cherry, Christopher Hamilton-West, Pedro Jimenez-Bluhm

**Affiliations:** 1 Escuela de Medicina Veterinaria, Facultad de Ciencias Biológicas, Facultad de Medicina, Facultad de Agronomía e Ingeniería Forestal, Pontificia Universidad Católica de Chile, Santiago 7820436, Chile; 2 Departamento de Medicina Preventiva Animal, Facultad de Ciencias Veterinarias y Pecuarias, Universidad de Chile, Santiago 8330111, Chile; 3 Laboratory for Analysis of the Biosphere (LAB), Universidad de Chile, Santiago 8330111, Chile; 4 Department of Infectious Diseases, St. Jude Children’s Research Hospital, Memphis, TN 38105, USA

**Keywords:** avian influenza, influenza A, Chile, remote sensing, Normalized Difference Vegetation Index (NDVI)

## Abstract

The Lluta River is the northernmost coastal wetland in Chile, representing a unique ecosystem and an important source of water in the extremely arid Atacama Desert. During peak season, the wetland is home to more than 150 species of wild birds and is the first stopover point for many migratory species that arrive in the country along the Pacific migratory route, thereby representing a priority site for avian influenza virus (AIV) surveillance in Chile. The aim of this study was to determine the prevalence of influenza A virus (IAV) in the Lluta River wetland, identify subtype diversity, and evaluate ecological and environmental factors that drive the prevalence at the study site. The wetland was studied and sampled from September 2015 to October 2020. In each visit, fresh fecal samples of wild birds were collected for IAV detection by real-time RT-PCR. Furthermore, a count of wild birds present at the site was performed and environmental variables, such as temperature, rainfall, vegetation coverage (Normalized Difference Vegetation Index—NDVI), and water body size were determined. A generalized linear mixed model (GLMM) was built to assess the association between AIV prevalence and explanatory variables. Influenza positive samples were sequenced, and the host species was determined by barcoding. Of the 4349 samples screened during the study period, overall prevalence in the wetland was 2.07% (95% CI: 1.68 to 2.55) and monthly prevalence of AIV ranged widely from 0% to 8.6%. Several hemagglutinin (HA) and neuraminidase (NA) subtypes were identified, and 10 viruses were isolated and sequenced, including low pathogenic H5, H7, and H9 strains. In addition, several reservoir species were recognized (both migratory and resident birds), including the newly identified host Chilean flamingo (*Phoenicopterus chilensis*). Regarding environmental variables, prevalence of AIV was positively associated with NDVI (OR = 3.65, *p* < 0.05) and with the abundance of migratory birds (OR = 3.57, *p* < 0.05). These results emphasize the importance of the Lluta wetland as a gateway to Chile for viruses that come from the Northern Hemisphere and contribute to the understanding of AIV ecological drivers.

## 1. Introduction

Considering that all influenza A virus (IAV) subtypes have the potential to contribute to the emergence of a pandemic strain, knowledge of infection dynamics and surveillance in wild birds is central to prevent the disease in humans and food-producing animals [1,2]. Wild waterfowl, particularly the Anseriformes and Charadriiformes orders, are considered main reservoirs of IAV in its low pathogenic form (LPAI) [1,3]. These birds have the potential to spread IAV when they migrate within and between continents, representing a risk for the emergence of highly pathogenic avian influenza (HPAI) outbreaks in domestic birds [4,5]. However, while much is known about IAV prevalence in Eurasian and North American wild birds [5,6,7,8], there remains a lack of information on IAV prevalence in wild birds from South America [9,10,11,12,13].

Chile has a coastline of more than 4000 km, along which there are hundreds of wetlands that serve as breeding grounds for resident birds and wintering areas for the many migratory species that arrive each year on the Pacific and Atlantic flyways [1,14]. Recent studies carried out by our research group have demonstrated the presence of a wide diversity of IAV subtypes in wild birds including low pathogenic H5 and H7 strains [9]. Sporadic outbreaks of LPAI have also been found in commercial farms and a widespread circulation of IAV has been found in backyard production systems (BPSs), which could be the result of spillover events from wild birds [15,16,17].

Along the Chilean coastline, there is a wide diversity of climates, which determines the environmental characteristics of each wetland. For example, the Lluta River wetland in Arica and Parinacota Region has a desert climate (<1 mm yearly rainfall); the El Yali wetland in the Valparaíso Region (central zone) has a climate comprising a cloudy steppe and warm temperate steppe with winter rainfall,; and the Itata River in Biobio Region (southern zone) has a warm temperate climate with dry and rainy seasons. This environmental variety of Chilean wetlands and the size of the country may affect the structure and composition of bird assemblages; however, this is still poorly studied in Chile, especially in the northern zone [18,19].

The coastal wetlands of northern Chile, being important sources of water in an extremely arid area, are ecosystems that shelter and serve as refuge for many species of wild birds [19]. The Lluta River wetland is the northernmost coastal wetland in Chile and belongs to the biogeographical province of the Coastal Desert. Its main water source is the Lluta River, the only exoreic river in the province, which experiences its most important floods during the summer season (December to March) due to the “highland winter” rains. The average annual temperature fluctuates between 16° and 22 °C and rainfall is almost non-existent throughout the year, reaching an annual maximum of 1.5 mm [19,20].

This wetland represents the main habitat of the local avifauna in the Atacama Desert and the gateway to the country for many migratory species that use the Pacific route [19,21]. The movements of those species may play an important role in the maintenance and spread of North American lineage viruses; therefore, this wetland may be a possible point of confluence where North and South American IAVs intermix [9], thereby representing a priority site for avian influenza virus surveillance in Chile.

However, to understand the role of wild birds in the spread and persistence of IAV, surveillance efforts must focus not only on the isolation and characterization of circulating viruses at sites where these birds concentrate; it is also critical that environmental factors that may impact IAV prevalence in these ecosystems be considered [22]. These factors include abiotic elements, such as rainfall and temperature; biotic factors, such as bird species present and amount of vegetation; and anthropogenic factors, such as land use and land cover types [22,23,24,25,26]. However, to date, studies that include ecological aspects of the virus and that explore the interaction between IAV prevalence in wild birds and the environment remain scarce [12,22,24,25,27]. So far, the only study performed in Chile that explores ecological and environmental factors related to the prevalence of IAV in wild birds demonstrated that the prevalence of IAV in central Chilean wetlands is not constant over the year, being higher in the summer and fall months. Environmental factors driving this prevalence included minimum temperature, Normalized Difference Vegetation Index (NDVI), and size of the wetland water body [12].

The aim of this study was to determine the prevalence of IAV in the Lluta River wetland between September 2015 and October 2020, identify subtype diversity, and evaluate ecological and environmental factors that drive these variables at the study site.

## 2. Materials and Methods

### 2.1. Study Area

The Lluta wetland (S 18°24′55″–W 70°19′20″) geographically belongs to the Lluta River watershed, the only exoreic river in the province of Arica in the Region of Arica and Parinacota. It is located on the coastal border, 10 km north of downtown Arica and 8 km from the border with Peru. It has an area of approximately 300 ha, of which only 31 ha is protected under the denomination of “Municipal Nature Reserve”. For birds, it is the most important wetland in northern Chile, hosting more than 150 species (both resident and migratory), which represent 32% of the bird species described for the country [19,20,21]. In 2010, it was designated a shorebird reserve by the Western Hemisphere Shorebird Reserve Network (WHSRN), a conservation initiative for the protection of the ecological integrity of critical habitats for shorebirds throughout the Americas [20].

This wetland corresponds to the first stopover point for many migratory species that arrive in the country along the Pacific migratory route, such as Franklin’s gull (*Leucophaeus pipixcan*), Whimbrel (*Numenius phaeopus*), Least sandpiper (*Calidris minutilla*), Baird’s sandpiper (*Calidris bairdii),* Lesser Yellowlegs (*Tringa flavipes*), and Greater Yellowlegs (*Tringa melanoleuca)*, which congregate in high densities during the spring and summer months [14,19,21].

### 2.2. Sampling and Sample Size

Sampling was carried out between September 2015 and October 2020, with a total of 19 sampling campaigns performed during that period. Samples were collected at irregular intervals. In each visit, fresh fecal samples of wild birds were collected for IAV detection. To minimize the probability of sampling the same individual’s feces twice, samples were collected uniformly by line transects throughout the area where a flock was previously observed. Limited samples were taken per each flock, with several sampling points in the site [12]. Samples were collected using single-use sterile flocked swabs (Copan^®^, Brescia, Italy) and stored in tubes containing 1 mL of universal transport medium (UTM, Copan^®^, Brescia, Italy). Samples were maintained at 4 °C until arrival at the laboratory of Veterinary Epidemiology of the School of Veterinary Sciences of the University of Chile. Samples were stored at −80 °C until processing. A minimum sample size of 178 samples per sampling occasion was calculated and estimated from the sample size calculation formula for detecting the presence of disease for finite populations (Equation (1)) [28]. A population size of 1000 fresh feces at the site was considered. In addition, based on results from a previous study [9], a minimum expected prevalence of 1.5% with a significance level of 5% was considered. 

Equation (1):

n=(1−(α)1D)(N−D−12),

where:

*n* = required sampling size;
*N* = population size;
α = 1-confidence level;
*D* = estimated minimum number of positive samples.

### 2.3. Influenza A virus Detection

The collected samples were processed individually, and RNA extraction and real-time RT-PCR (RT-qPCR) analysis were performed at the Laboratory of Veterinary Epidemiology. Briefly, viral RNA extraction was performed on 50 μL of swab sample using the Ambion MagMax-96 AI/ND viral isolation kit (Life Technologies Corporation, Grand Island, NY, USA). Sample screening was performed using RT-qPCR (Stratagene mx3000 p Santa Clara, CA, USA) with the TaqMan Fast Virus 1-Step Master Mix (Applied Biosystems, Foster City, CA, USA). Specific primer/probes for the influenza M gene were used for the RT-qPCR reaction as previously described [29]. Samples with a cycle threshold value (Ct) ≤ 38 were considered positive [30]. Swab samples that tested positive by RT-qPCR with a Ct ≤ 35 were inoculated in embryonated chicken eggs to virus isolation as previously described [31]. Host identification was attempted for IAV-positive fecal samples using primers designed to amplify a segment of the mitochondrial cytochrome-oxidase I as described elsewhere [32].

### 2.4. Sequencing and Phylogenic Analysis

Sample sequencing was carried out at the St. Jude Children’s Hospital Hartwell Center on the Illumina MiSeq sequencer platform (Illumina, San Diego, CA, USA). Libraries were prepared using the Nextera XT DNA-Seq library preparation kit, as previously described [33]. Reads were de novo assembled using the SAPDes package [34]. Reference sequences for the phylogenetic analysis were obtained from the Influenza Virus Database [35]. Representative sequences from North America, Europe, and Asia were included in each tree. Briefly, sequence editing was performed using BioEdit version 7.2.5 [36] and sequence alignment was performed with MUSCLE version 3.8.3 [37]. Phylogenetic inference was undertaken for the HAs of the H5, H7, and H9 isolates by maximum likelihood, with a general time-reversible model and gamma-distributed rate variation among sites. Maximum likelihood analysis was performed using RAxML version 8.0 [38]. To provide statistical robustness of each node, 1000 bootstrap resampling processes were performed. Trees were visualized on FigTree (v.1.4.3). Genbank accession numbers are KX185901, KX185918, MH499035, MH498968, MH498978, MH499057, MH499142, MK164009, OP888556, and OQ820949.

### 2.5. Ecological and Environmental Variables

The ecological and environmental variables evaluated in this study were grouped into three categories (Table 1).

**Wild bird community:** At each visit and prior to sampling, a count of wild birds present at the site was performed during morning hours. A point-counting approach with several experienced observers was used [12]. These data were used to estimate total bird abundance, species richness, and abundance of migratory birds present at the time of sampling.

**Landscape variables**: The vegetative cover of the wetland and the size of the water body was measured for each sampling month, using images of the LANDSAT 7-ETM and 8-OLI satellites. In addition, to assess the cumulative effect of landscape characteristics and IAV prevalence at the site, these variables were also collected one month, two months, and three months before the sampling month [12]. Satellite images were downloaded from the website of the United States Geological Survey (USGS EarthExplorer at https://earthexplorer.usgs.gov/) and processed using ENVI^®^ software version 4.7. The Normalized Difference Vegetation Index (NDVI) was used as an indicator of the vegetation cover; to delineate water bodies, the Modified Normalized Difference Water Index (MNDWI) was used [39,40].

**Meteorological data**: Monthly average temperatures, humidity, and cumulative rainfall were measured for each sampling month. To investigate the cumulative effect of rainfall, humidity, and temperature on IAV prevalence, these variables were also collected one month, two months, and three months beforehand [12]. The information was obtained from the Lluta Bajo weather station, which belongs to Agrometeorological Network (Agromet) of the Agricultural Research Institute (INIA) (https://agrometeorologia.cl/).

### 2.6. Statistical Analysis

To determine the association between the prevalence of IAV in the wetland and the ecological and environmental explanatory variables defined in Table 1, a GLMM was used. The response variable was defined as the number of IAV-positive samples obtained in each sampling in relation to sample size (prevalence). The sampling occasion was entered as a random effect in the model [12]. The unconditional association between IAV prevalence and each of the recorded explanatory variables was assessed in the first bivariate GLMM (Supplemental Table S1). Variables associated with the outcome at a liberal *p*-value of <0.15 were selected for inclusion in the multivariable model.

The linearity of continuous explanatory variables against the log odds of IAV positivity was assessed visually. Non-linear variables were categorized using the median. A forward stepwise inclusion of variables, guided by the Akaike Information Criterion (AIC), was performed to build the final multivariable mixed model. All analyses were performed using R statistical software using the “glmer” function of the “lme4” package [41]. The significance level was set at 5%.

## 3. Results

### 3.1. Influenza Prevalence and Species Richness

Between September 2015 and October 2020, a total of 4349 fecal samples were collected, of which 90 (2.07%, 95% CI: 1.68 to 2.55) were positive for the influenza virus M gene according to RT-qPCR. Thirty-five of these samples were inoculated on eggs from which the isolates were obtained (Supplemental Table S2).

**Figure 1:**
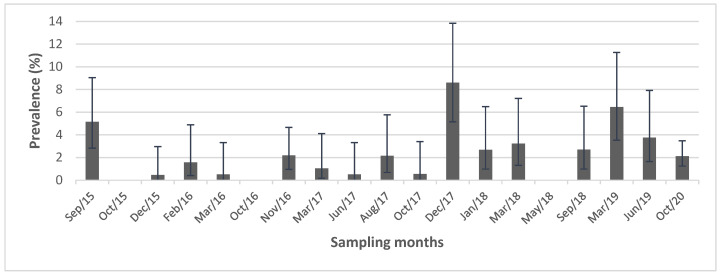
Prevalence (95% CI) of influenza A virus in the Lluta wetland during the sampling months.

Over the five years of sampling, the prevalence of IAV varied widely (between 0% and 8.6%), showing apparent peaks during the months when wild migratory birds were present (Figure 1). However, because sampling was carried out at irregular intervals, it was not possible to determine seasonality in this prevalence.

**Figure 2:**
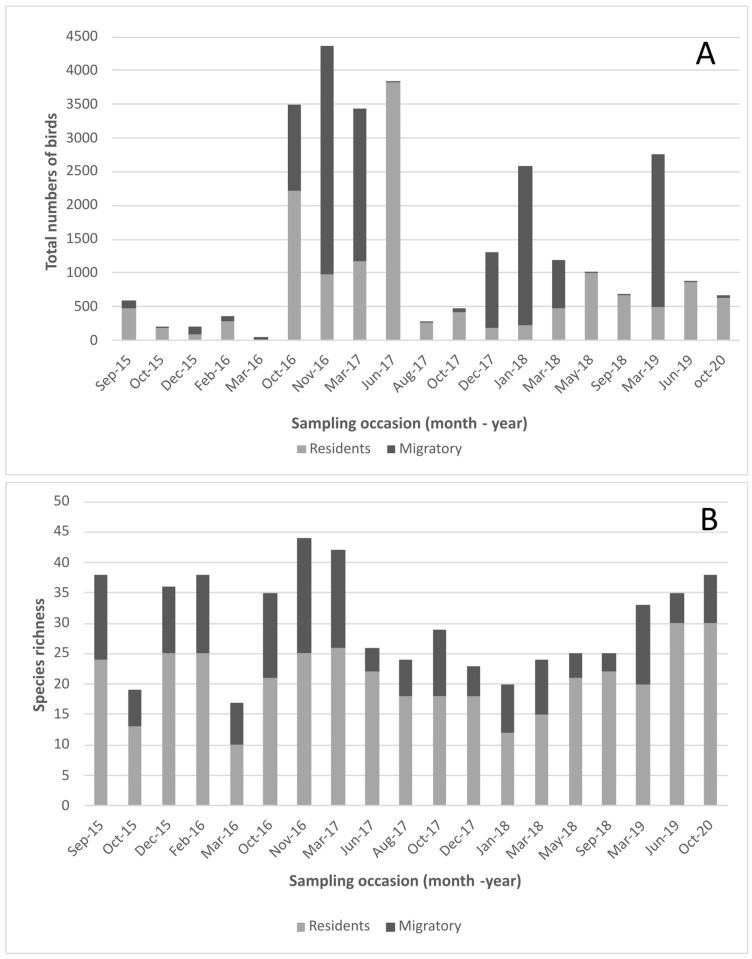
Total number of birds counted (**A**) and species richness (**B**) identified during the sampling occasions (month–year) in the Lluta River wetland.

Bird numbers fluctuated widely among sampling occasions over the study period (Figure 2A), where a greater number of individuals were observed during the summer and early autumn months, mainly due to the arrival of migratory birds at the site, highlighting species such as Franklin’s gull (*Leucophaeus pipixcan*), Whimbrel (*Numenius phaeopus*), and many species of shorebirds.

Species richness observed during the study months ranged from a minimum of 17 to a maximum of 44, showing an increase during the spring and summer months with the arrival of migratory species from the Northern Hemisphere (Figure 2B). During the study period, a total of 71 species were identified in the wetland, of which 41 are considered resident, 21 migratory, and 9 accidental (Supplemental Table S3).

### 3.2. Environmental Variables

Meteorological variables (precipitation, humidity, maximum and minimum temperatures) did not show large variations during the months of study (Supplemental Table S4) and none of them showed significance with the prevalence of IAV in the wetland.

**Figure 3:**
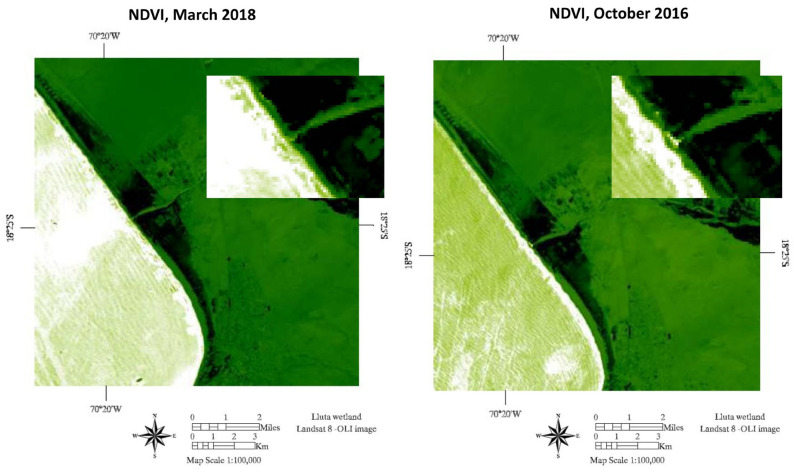
NDVI images in Lluta wetland in the months of highest (March 2018) and lowest (October 2016) vegetation cover values during the study period.

Regarding environmental variables, the amount of water in the wetland increased between January and March and reached its lowest level between October and December, while the vegetation cover (measured by NDVI) was higher between February and March and reached its lowest level in the months of October and November. Figure 3 shows the months in which the greatest variations in NDVI (lowest and highest) occurred during the study period.

The final multivariable model showed that IAV prevalence was positively associated with NDVI for the month of sampling and with the abundance of migratory birds (Table 2). The months in which 113 or more individuals belonging to migratory species were counted were associated with higher prevalence (OR = 3.57, *p* < 0.05). With respect to vegetation, the months in which NDVI values were equal to or greater than 0.27 were associated with higher prevalence (OR = 3.65, *p* < 0.05).

None of the variables included in the “Meteorological data” category were retained in the final multivariable model.

### 3.3. Subtype Diversity and Sequence Analysis

**Figure 4:**
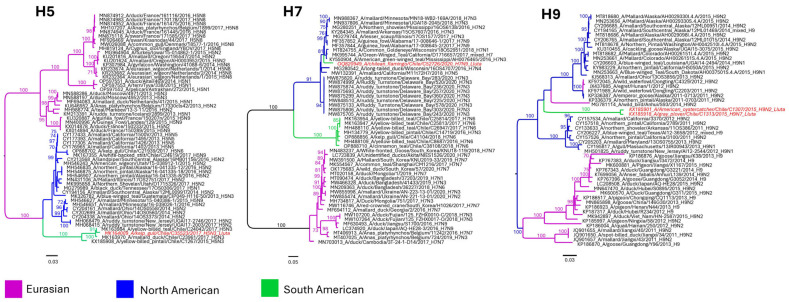
Maximum likelihood phylogenetic analysis of the hemagglutinins H5, H7, and H9. North American clade in blue, Eurasian clade in purple, and South American clade in green. Study sequences in red and italic. Bootstrap support > 70% indicated.

A wide diversity of hemagglutinin (HA) and neuraminidase (NA) subtypes were identified, and 10 viruses were isolated, including low pathogenic H5 and H7 strains (Table 3). Six reservoir species were recognized, including *Larus dominicanus* (Kelp gull, *n* = 2), *Haematopus palliatus* (American oystercatcher, *n* = 3), *Haematopus ater* (Blackish oystercatcher, *n* = 1), *Larus pipixcan* (Franklin’s gull, *n* = 2), *Pluvialis squatarola* (Grey plover, *n* = 1), and newly identified host *Phoenicopterus chilensis* (Chilean flamingo, *n* = 1). All obtained viruses were LPAI according to HA cleavage sequence analysis. Results of the phylogenetic analysis performed on H5, H7, and H9 hemagglutinins (Figure 4), revealed that the H5 hemagglutinin was most like other H5 hemagglutinins obtained in the central region of Chile between 2015 and 2017. Conversely, the H7 hemagglutinin belonged to the North American clade of viruses, with similar sequences obtained from birds belonging to the Mississippi and Pacific flyways. Additionally, H9 viruses did not form a distinct clade within the global H9 phylogeny but were closely related to each other and to other isolates obtained in North America.

## 4. Discussion

This study provides information on the prevalence of IAV and the environmental factors that could influence its occurrence in a wetland of great importance for IAV surveillance in Chile. In addition, this wetland has been a key site for surveillance of the current HPAI H5N1 2.3.4.4b outbreak that is affecting several countries in South America, and is the site where the first positive case was detected in Chile in December 2022 [42,43]. Therefore, our results provide important considerations for structuring surveillance and early warning actions in a wetland that is the gateway to the viruses from North America.

The overall IAV prevalence identified at the Lluta wetland during the months sampled was 2.07% (95% CI: 1.68–2.55), which is lower than that recorded in sites in the central zone of Chile during the same study period (4.28%) [12]. This could be due to the difference between the bird assemblages in both areas. While in Lluta wetland the predominant species correspond to shorebirds, in the wetlands of central Chile there are many resident ducks that have already been identified as primary hosts of IAV viruses [9] and may have an important role in the perpetuation of the virus throughout the year [12]. In another previously published study that included sampling in the Lluta wetland, the prevalence found was also higher (5.15%); however, the aforementioned prevalence corresponds to a single sampling event [9].

Regarding subtype diversity and origins, this is the first time that an H7 virus originating from North America has been found in the northern part of Chile, and its presence may have significant implications. Unlike the South American clade of H7 viruses, which has only been associated with HPAI in South America once [44], North American strains have been responsible for numerous outbreaks of HPAI [45,46]. On the other hand, the H5 and H9 HAs appear to be of South American origin, although further genomic surveillance is necessary to definitively establish their clade formation.

With respect to the ecological variables evaluated in this study, only the abundance of migratory birds was positively related to the prevalence of IAV at the site. None of the other variables in the “bird community” category (species richness and total bird abundance) showed statistical association with prevalence. This is probably because bird abundance also increased in months where prevalence was low (autumn and winter), due to large numbers of birds that are considered resident at the site, such as the Grey gull (*Leucophaeus modestus*). On the other hand, although species richness has been considered a factor influencing the prevalence of the virus in studies carried out in Spain [25,27], other studies around the world have found that bird density or the abundance of migratory species have a greater influence on the prevalence of AIV than bird richness [22,47,48], which is in agreement with the present results obtained in Lluta wetland.

The number of migratory birds in the Lluta River wetland increases considerably between October and March, with high densities at the site, and then declines to almost zero during the rest of the year. However, there are variations in the abundance of migratory birds detected in the same months between different years (Figure 2A). This could be explained because migratory patterns are driven by environmental conditions (such as temperature, food availability, and landscape structure) [49,50], which may vary from year to year or be influenced by climate change [51] or by climatic phenomena such as the El Niño–Southern Oscillation (ENSO), the main driver of interannual climate extremes in South America [52].

The species of migratory birds identified belong to the families Laridae, Scolopacidae, and Charadriidae, which have been identified as important reservoirs of IAV [1,3]. Franklin’s gull (*Leucophaeus pipixcan*), Whimbrels (*Numenius phaeopus*), and Sanderling (*Calidris alba*) were the species with the highest number of individuals during the sampling period (Supplemental Table S1). In addition, it is important to highlight that three viral isolates in our study were found in migratory species: two in Franklin’s gulls (H13N9) and one H9N7 subtype in a Grey plover (*Pluvialis squatarola*). Recent studies in Chile and Peru have already described the infection of IAV in Franklin’s gulls [9,53,54], Grey plover [9], and Whimbrels [9,10,55]. In one of these studies, which determined a wide diversity of IAVs in wetlands in Chile, it was also found that the viruses isolated in Lluta were the most genetically diverse, with segments of viruses from North American and South American lineages [9], confirming the thesis of the importance of migratory birds in the dissemination of IAV and in the emergence of new subtypes.

With respect to the meteorological variables evaluated in this study, none were significantly associated with IAV positivity to the virus in the wetland, although climatic variables such as temperature and precipitation have been described as important drivers of IAV in wild birds in other regions of the world [24,26,56,57]. This could be explained mainly because climatic conditions in the wetland are stable throughout the year. Precipitation in the area is almost non-existent and the average annual temperature is around 18 °C [21]. The same is observed in wetlands in tropical Africa, where climatic factors were poorly related to IAV prevalence in wild birds, but host ecological factors (such as bird density in wetlands and the arrival of migrants from Eurasia) played a much more important role [22]. This highlights the relevance of conducting studies that include environmental factors in a local context.

Regarding landscape variables, vegetative coverage in the sampling month (measured by NDVI) was positively associated with the prevalence of IAV in the wetland. During the study period, NDVI values fluctuated between 0.18 and 0.31, which is related to the presence of shrub and herbaceous vegetation [58] consisting mainly of communities of horsetail, chilca shrubs, salt grass, and reed [19]. The months in which NDVI values were equal to or greater than 0.27 were associated with higher prevalence. These results are similar to those found in Europe, where vegetation surrounding wetlands has been recognized as an important driver in the presentation dynamics of the IAV [25,26], because wild birds are impacted by the availability of food resources and shelter provided by a wetland. Higher NDVI may indicate the presence of better vegetative food resources and shelter, favoring a higher concentration of birds and therefore a higher risk of pathogen transmission [25,59]. In another study conducted in the Poyang Lake wetland in China, it was determined that there is a positive correlation coefficient between NDVI and the number of birds present in the wetland, especially migratory birds [59]. In the Lluta wetland, NDVI reaches its maximum between October and March. These months coincide with the arrival of migratory birds from the Northern Hemisphere and with a greater growth of vegetation in spring, which extends into the summer mainly due to the increase in the flow of the Lluta River due to the “highland winter” rains (December to March).

With respect to water size, this variable was not significant in the prevalence of AIV in the Lluta wetland. These results were different from those found in central Chile where the extreme variations in the water body size experienced by the wetlands during summer months directly affect the congregation of birds and therefore the dynamics of infection of the IAV [12]. During the study period, the Lluta wetland did not show significant variations in the surface area flooded by water. Only during January 2018 was a larger area of water observed in the wetland compared to the other months sampled. However, because the area of the wetland that is flooded with water is relatively small (during the sampling months it fluctuated between 0.5 and 2 ha), the concentration of hosts in those areas, especially during the times of migrant arrivals, generates high densities of individuals favoring the increase in prevalence at the site due to density-dependent transmission of the agent. This is similar to what is observed in wetlands in tropical Africa [60].

With respect to the IAV host species identified, in addition to the two migratory species mentioned above, IAV isolates were found in American and blackish oystercatcher (*Haematopus palliates, Haematopus ater*), Kelp gull (Larus dominicanus), and a Chilean flamingo (Phoenicopterus chilensis). Although the first three species have already been described as hosts in other studies in Chile and South America [9,10,11,61,62], this is the first time that IAV has been isolated in a Chilean flamingo. This species corresponds to a neotropical migrant that is widely distributed throughout South America, even reaching the Atlantic coast [63]. Therefore, it may be an important species for the spread of IAV between South American countries.

Although our results contribute to understanding the ecological drivers of IAV in the Lluta River wetland, the study had some limitations. Because sampling was conducted at irregular intervals, it was not possible to determine a seasonal pattern in this prevalence. Sampling was carried out in this way, because the Lluta wetland is in a remote area, where, for logistical reasons, it was not possible to carry out a more systematic sampling. In addition, the last year of the study (2020) coincided with the SARS-CoV-2 pandemic. Therefore, we performed only one sampling during that year.

However, due to the epidemiological importance of this wetland for IAV surveillance in Chile, it would be beneficial to extend this long-term longitudinal study. Ideally, the study would include a greater number of samples at regular intervals to determine whether there is a seasonal pattern in the prevalence of the virus at the site. In addition, this would allow us to understand the difference in prevalence found in the same months between different study years and would allow a better evaluation of the risk associated with the transmission of the virus from wildlife reservoirs to domestic birds. This would also improve surveillance programs by directing sampling efforts towards seasons that are more favorable for the maintenance and transmission of the virus.

## 5. Conclusions

Our results demonstrate that there is a wide circulation of IAV in wild birds of the Lluta River wetland and a wide range of different influenza subtypes present in the bird populations, including ones considered as “high-risk”. Ten influenza viruses were isolated and sequenced, and this is the first time that an H7 virus having a North American origin was found in the northern part of Chile.

The prevalence of IAV was positively associated with NDVI and with the abundance of migratory birds in the wetland. However, additional long-term studies are needed to include other host-related variables (such as age structure of the population and density of individuals) and to evaluate whether there is a seasonal pattern of prevalence in this wetland.

Our results constitute a unique contribution to the understanding of the ecological drivers that may modulate the occurrence of IAV in northern Chile, providing important considerations for the global surveillance of IAV in wild birds and highlighting the importance of the Lluta wetland as a gateway to Chile for viruses that come from the Northern Hemisphere.

## Figures and Tables

**Table 1:** Definition of ecological and environmental variables.

Variables		Definition
**Wild bird community**	Total abundance	Total number of birds present at the time of sampling
	Species richness	Number of species present at the time of sampling
	Abundance of migrants	Number of migratory birds present at the time of sampling
**Landscape**	Vegetation coverage	Mean NDVI for the month of sampling, one month before, two months before, and three months before sampling.
	Water body size (km^2^)	Water body size for the month of sampling, one month before, two months before, and three months before sampling.
**Meteorological data**	Maximum monthly temperature (°C)	Monthly mean of maximum daily temperature for the month of sampling, one month before, two months before, and three months before sampling.
	Minimum monthly temperature (°C)	Monthly mean of minimum daily temperature for the month of sampling, one month before, two months before, and three months before sampling.
	Total monthly rainfall (mm)	Total rainfall at the month of sampling, one month before, two months before, and three months before sampling.
	Humidity (%)	Relative air humidity at the month of sampling, one month before, two months before, and three months before sampling.

**Table 2:** Multivariable model results of the association between IAV prevalence and environmental variables.

Variables	Categories	Estimate	*p*-Value	OR	95% CI
**(Intercept)**		−5.5872	<0.001	0.003	(0.001–0.01)
**NDVI**	Low (<0.27)	Reference			
	High (≥0.27)	1.2949	0.00230	3.65	(1.58–8.39)
**Abundance of Migrants**	Low (<113)	Reference			
	High (≥113)	1.2727	0.00733	3.57	(1.41–9.05)

IAVs isolated from wild birds in and hosts species.

**Table 3:** IAV strain name by year, subtypes, host species, and GenBank accession of IAVs identified in the Lluta River wetland between September 2015 and October 2020.

Strain Name	Year	Isolate Subtype	Host Species	Order	Accession Number
A/American oystercatcher/Chile/C1307/2015	2015	H9N2	*Haematopus palliatus*	Charadriiformes	KX185901
A/Grey plover/Chile/C1313/2015	2015	H9N7	*Pluvialis squatarola*	Charadriiformes	KX185918
A/American oystercatcher/Chile/C17359/2016	2016	H3N8	*Haematopus palliatus*	Charadriiformes	MH499035
A/American oystercatcher/Chile/C17400/2016	2016	H3N8	*Haematopus palliatus*	Charadriiformes	MH498968
A/Franklin’s gull/Chile/C17421/2016	2016	H13N9	*Larus pipixcan*	Charadriiformes	MH498978
A/Franklin’s gull/Chile/C17422/2016	2016	H13N9	*Larus pipixcan*	Charadriiformes	MH499057
A/Kelp gull/Chile/C27733/2017	2017	H13N8	*Larus dominicanus*	Charadriiformes	MH499142
A/Kelp gull/Chile/C35523/2017	2017	H5N3	*Larus dominicanus*	Charadriiformes	MK164009
A/blackish oystercatcher/Chile/C40194/2018	2018	H13N2	*Haematopus ater*	Charadriiformes	OP888556
A/Chilean flamingo/Chile/C52796/2020	2020	H7N9	*Phoenicopterus chilensis*	Phoenicopteriformes	OQ820949

## Data Availability

The data presented in this study are available on request from the corresponding author.

## Supplementary Materials

The following supporting information can be downloaded at: https://www.mdpi.com/article/10.3390/v15061241/s1. Supplemental Table S1: Bivariable associations between IAV prevalence and explanatory variables; Supplemental Table S2: Sampling effort detail. Total number of samples collected in the study period, number of positive samples, samples inoculated in eggs and successfully sequenced; Supplemental Table S3. Species identified in the Lluta River wetland between September 2015 and October 2020; Supplemental Table S4: Ranges of meteorological and landscape variables during the sampling months.
