# Supplementary material for: Remote Sensing and Ecological Variables Related to Influenza A Prevalence and Subtype Diversity in Wild Birds in the Lluta Wetland of Northern Chile
Source: Viruses. 2023 May 25;15(6):1241. doi: 10.3390/v15061241 (PMC10303387; doi:10.3390/v15061241)
Supplement: Supplementary file 1 [file viruses-15-01241-s001.zip › viruses-2406292-supplementary.pdf]

## Supplemental Material:

## Supplemental Table S1: Bivariable associations between IAV prevalence and explanatory variables

| Variables | Categories | Estimate | p-value | OR | 95% CI |
|---|---|---|---|---|---|
| **Abundance of wild birds** | Low (< 883) | Reference | | | |
| | High (≥ 883) | 0.45 | 0.365 | 1.57 | (0.59-4.13) |
| **Species richness** | Low (< 29 sp) | Reference | | | |
| | High (≥ 29 sp) | 0.15 | 0.764 | 1.17 | (0.42-3.21) |
| **Abundance of migrants** | Low (< 113) | Reference | | | |
| | High (≥ 113) | 0.8135 | 0.045** | 2.26 | (1.04 -5.74) |
| **NDVI** | Low (< 0.27) | Reference | | | |
| | High (≥ 0.27) | 1.23 | 0.0264** | 3.44 | (1.15- 8.24) |
| **NDVI 1 month bs** | Low (< 0.27) | Reference | | | |
| | High (≥ 0.27) | -0.3195 | 0.617 | 0.73 | (0.21-2.53) |
| **NDVI 2 month bs** | Low (< 0.27) | Reference | | | |
| | High (≥ 0.27) | -0.1149 | 0.901 | 0.89 | (0.14- 5.41) |
| **NDVI 3 month bs** | Low (< 0.27) | Reference | | | |
| | High (≥ 0.27) | -0.6823 | 0.429 | 0.59 | (0.15- 3.21) |
| **Water body size (km2)** | Low (< 0.014) | Reference | | | |
| | High (≥ 0.014) | 0.6600 | 0.31 | 1.93 | (0.54 -6.92) |
| **Water body size 1 month bs** | Low (< 0.018) | Reference | | | |
| | High (≥ 0.018) | 0.8250 | 0.167 | 2.28 | (0.71 -7.35) |
| **Water body size 2 month bs** | Low (< 0.016) | Reference | | | |
| | High (≥ 0.016) | 0.4756 | 0.607 | 161 | (0.26- 9.85) |
| **Water body size 3 month bs** | Low (< 0.013) | Reference | | | |
| | High (≥ 0.013) | -0.2155 | 0.687 | 0.81 | (0.28- 2.29) |
| **Maximum temperature (°C)** | Low (< 26) | Reference | | | |
| | High (≥ 26) | -0.09148 | 0.859 | 0.92 | (0.33- 2.50) |
| **Maximum temperature 1 month bs** | Low (< 23) | Reference | | | |
| | High (≥ 23) | -0.4908 | 0.323 | 1.14 | (0.42- 3.11) |
| **Maximum temperature 2 month bs** | Low (< 24) | Reference | | | |
| | High (≥ 24) | 0.4483 | 0.37 | 1.56 | (0.58- 4.17) |
| **Maximum temperature 3 month bs** | Low (< 20) | Reference | | | |
| | High (≥ 20) | 0.2514 | 0.623 | 1.29 | (0.47- 3.51) |
| **Minimum temperature (°C)** | Low (< 13.5) | Reference | | | |
| | High (≥ 13.5) | 0.4812 | 0.367 | 1.62 | (0.56 -4.59) |
| **Minimum temperature 1 month bs** | Low (< 14.2) | Reference | | | |
| | High (≥ 14.2) | 0.1340 | 0.793 | 1.14 | (0.42 -3.11) |
| **Minimum temperature 2 month bs** | Low (< 13.4) | Reference | | | |

| | | | | | |
|---|---|---|---|---|---|
| | High (≥ 13.4) | -0.3620 | 0.474 | 0.69 | (0.26- 1.87) |
| **Minimum temperature 3 month bs** | Low (< 14.5)<br>High (≥ 14.5) | Reference<br>0.2011 | 0.695 | 1.28 | (0.47 -3.51) |
| **Humidity (%)** | Low (< 61.5)<br>High (≥ 61.5) | Reference<br>-0.6116 | 0.224 | 0.54 | (0.203-1.45) |
| **Humidity 1 month bs** | Low (< 61.9)<br>High (≥ 61.9) | Reference<br>0.2384 | 0.645 | 1.27 | (0.46-3.50) |
| **Humidity 2 month bs** | Low (< 62.7)<br>High (≥ 62.7) | Reference<br>0.1391 | 0.792 | 1.15 | (0.41-3.24) |
| **Humidity 3 month bs** | Low (< 62.7)<br>High (≥ 62.7) | Reference<br>0.1162 | 0.823 | 1.12 | (0.41-3.11) |

**Supplemental Table S2: Sampling effort detail. Total number of samples collected in the study period, number of positive samples, samples inoculated in eggs and successfully sequenced.**

| Number of samples collected | Number of samples positive for M gene (Ct<38) | Number of samples inoculated in eggs (Ct<35) | Number of isolates | Number of completely sequenced isolates |
|---|---|---|---|---|
| 4349 | 90 | 35 | 16 | 10 |

**Supplemental Table S3. Species identified in the Lluta River wetland between September 2015 and October 2020.**

| Family / Bird species | Commun Name | Maximum count on site | Residency Status |
|---|---|---|---|
| **Anatidae** | | | |
| *Spatula cyanoptera* | Cinnamon teal | 36 | R |
| *Anas bahamensis* | White-cheeked pintail | 40 | R |
| *Anas georgica spinicauda* | Yellow-billed Pintail | 2 | R |
| *Spatula discors* | Blue-winged teal | 1 | A (M) |
| *Oxyura jamaicensis* | Andean duck | 1 | A |
| **Charadriidae** | | | |
| *Pluvialis dominica* | Golden plover | 25 | M |
| *Charadrius nivosus* | Snowy plover | 2 | R |
| *Charadrius semipalmatus* | Semipalmated Plover | 30 | M |
| *Charadrius vociferus peruvianus* | Killdeer | 10 | R |

| | | | |
|---|---|---|---|
| *Pluvialis squatarola* | Grey Plover | 8 | M |
| **Laridae** | | | |
| *Chroicocephalus serranus* | Andean gull | 350 | R |
| *Chroicocephalus maculipennis* | Brown-hooded gull | 300 | R |
| *Leucophaeus pipixcan* | Franklin gull | 2300 | M |
| *Larus dominicanus* | Kelp gull | 300 | R |
| *Larus belcheri* | Belcher's Gull | 60 | R |
| *Leucophaeus modestus* | Grey gull | 3000 | R |
| *Thalasseus elegans* | Elegant tern | 90 | M |
| *Larosterna inca* | Inca Tern | 25 | R |
| *Sterna hirundinacea* | South American tern | 3 | R |
| *Sternula antillarum* | Least Tern | 1 | A (M) |
| *Rynchops niger cinerascens* | Black Skimmer | 66 | M |
| **Scolopacidae** | | | |
| *Calidris canutus* | Red Knot | 1 | M |
| *Calidris alba* | Sanderling | 700 | M |
| *Calidris bairdii* | Baird's sandpiper | 8 | M |
| *Arenaria interpres* | Ruddy turnstone | 8 | M |
| *Calidris pusilla* | Semipalmated Sandpiper | 6 | M |
| *Calidris himantopus* | Stilt Sandpiper | 15 | M |
| *Calidiris minutilla* | Least sandpiper | 2 | M |
| *Tringa semipalmata* | Willet | 19 | M |
| *Actitis macularius* | Spotted sandpiper | 5 | M |
| *Calidris melanotos* | Pectoral sandpiper | 2 | M |
| *Phalaropus tricolor* | Wilson's Phalarope | 8 | M |
| *Numenius phaeopus* | Whimbrel | 500 | M |
| *Limosa haemastica* | Hudsonian Godwit | 16 | M |
| *Tringa flavipes* | Lesser Yellowlegs | 10 | M |
| *Tringa melanoleuca* | Greater Yellowlegs | 50 | M |
| **Haematopodidae** | | | |
| *Haematopus palliatus palliatus* | American oystercatcher | 123 | R |
| *Haematopus ater* | Blackish oystercatcher | 18 | R |
| **Ardeidae** | | | |
| *Egretta caerulea* | Little Blue Heron | 8 | R |
| *Bubulcus ibis ibis* | Cattle Egret | 1 | R |
| *Egretta thula thula* | Snowy egret | 30 | R |
| *Ardea alba egretta* | Great egret | 30 | R |
| *Egretta tricolor* | Tricolored Heron | 1 | A |
| *Nycticorax nycticorax* | Black-crowned Night-Heron | 84 | R |
| *Nyctanassa violacea* | Yellow-crowned Night-Heron | 1 | A |
| **Rallidae** | | | |
| *Fulica leucoptera* | White-winged Coot | 1 | R |

| | | | |
|---|---|---|---|
| *Fulica ardesiaca* | Slate-colored Coot | 20 | A |
| *Gallinula galeata* | Common Gallinule | 32 | R |
| **Pelecanidae** | | | |
| *Pelecanus thagus* | Peruvian Pelican | 60 | R |
| *Sula variegata* | Peruvian Booby | 20 | R |
| ***Threskiortnithidae*** | | | |
| *Plegadis chihi* | White-faced Ibi | 3 | R |
| *Platalea ajaja* | Roseate Spoonbill | 1 | A |
| *Plegadis ridgwayi* | Puna Ibis | 4 | A |
| **Phalacrocoracidae** | | | |
| *Phalacrocorax brasilianus brasilianus* | Neotropic Cormorant | 42 | R |
| **Falconidae** | | | |
| *Falco sparverius* | American Kestrel | 2 | R |
| *Falco peregrinus* | Peregrine falcon | 1 | R |
| ***Tyrannidae*** | | | |
| *Lessonia oreas* | Andean Negrito | 2 | R |
| *Lessonia rufa* | Austral Negrito | 2 | R |
| *Muscisaxicola maculirostris* | Spot-billed ground tyrant | 1 | R |
| *Muscisaxicola maclovianus* | Dark-faced ground tyrant | 1 | R |
| ***Troglodytidae*** | | | |
| *Troglodytes aedon* | House wren | 5 | R |
| ***Emberizidae*** | | | |
| *Zonotrichia capensis* | Rufous-collared Sparrow | 26 | R |
| ***Cathartidae*** | | | |
| *Cathartes aura jota* | Turkey Vulture | 94 | R |
| ***Icteridae*** | | | |
| *Leistes bellicosus* | Peruvian meadowlark | 8 | R |
| *Molothrus bonariensis* | Shiny Cowbird | 4 | R |
| ***Columbidae*** | | | |
| *Columbina cruziana* | Croaking Ground-Dove | 4 | R |
| *Zenaida meloda* | West Peruvian Dove | 23 | R |
| *Zenaida auriculata* | Eared Dove | 2 | R |
| ***Thraupidae*** | | | |
| *Conirostrum cinereum* | Cinereous Conebill | 17 | R |
| *Xenospingus concolor* | Slender-billed finch | 31 | R |
| ***Phoenicopteridae*** | | | |
| *Phoenicopterus chilensis* | Chilean Flamingo | 2 | A |

***Residency Status: R: Resident, M: Migratory, A: Accidental***

**Supplemental Table S4: Ranges of meteorological and landscape variables during the sampling months**

| Variables | Min | Max | Median |
|---|---|---|---|
| **Min. Temp (C°)** | 9 | 18 | 14 |
| **Max. Temp(C°)** | 20 | 30 | 26 |
| **Rainfall (mm)** | 0 | 3 | 0.37 |
| **Humidity (%)** | 55.7 | 68.30 | 61.50 |
| **NDVI** | 0.18 | 0.31 | 0.27 |
| **Water body size (km2)** | 0.0045 | 0.0333 | 0.014 |
